# The Use of Transperineal and Endoanal Ultrasound in Diagnosis and Management of Obstetric Anal Sphincter Injuries (OASIs): A Narrative Review from a Gynecologic Perspective

**DOI:** 10.3390/diagnostics16101458

**Published:** 2026-05-11

**Authors:** Christina Pagkaki, Panayiota Papasozomenou, Efthymios Oikonomou, Sofoklis Stavros, Anastasios Potiris, Nikoletta Koutlaki, Menelaos Zafrakas, Angeliki Gerede

**Affiliations:** 1 Department of Obstetrics and Gynecology, Democritus University of Thrace, 69100 Alexandroupolis, Greece; christinapagkaki@gmail.com (C.P.);; 2 School of Health Science, International Hellenic University, Sindos, 57400 Thessaloniki, Greece; 3 Department of Obstetrics and Gynecology, University General Hospital “ATTIKON”, Medical School, National and Kapodistrian University of Athens, 12462 Athens, Greeceapotiris@med.uoa.gr (A.P.)

**Keywords:** obstetric anal sphincter injuries (OASIs), transperineal ultrasound, endoanal ultrasound, narrative review

## Abstract

Obstetric anal sphincter injuries (OASIs) are serious complications of vaginal delivery and the most frequent cause of postpartum anal incontinence. Underdiagnosis during delivery persists due to limited availability of accurate imaging. Transperineal ultrasound (TPUS) and endoanal ultrasound (EAUS) enable structural assessment of the anal sphincter and have growing roles in triage, repair planning, and follow-up.The aim of the present review is to integrate current evidence on diagnostic value, clinical usefulness, and potential implementation of TPUS and EAUS in gynecologic practice.Ultrasound identifies a high burden of occult sphincter trauma in primiparas with normal clinical assessment with rates close to 25%. EAUS is currently the gold standard for accurate imaging of defects. TPUS has excellent diagnostic performance for external anal sphincter (EAS) defects and is practical for bedside use. Improving the diagnosis of OASIs requires structured post-delivery clinical examination combined with selective use of TPUS as a first-line imaging modality and EAUS in cases of diagnostic uncertainty or suspected complex injury.Immediate postpartum ultrasound may help reduce delayed diagnoses and support timely surgical repair. Key barriers include operator dependence, training, availability, and variable image quality, particularly for routine EAUS in the labor ward.Incorporating ultrasound into postpartum management improves detection and classification of OASIs and subsequently repair and prognosis. Currently, judicious application in high-risk deliveries seems to be an appropriate approach until more evidence from prospective and economic studies is available. New technologies (3D/4D imaging, standardized terminology, and decision-support/AI) hold promise to increase reproducibility and use in everyday clinical practice.

## 1. Introduction

**Figure 1:**
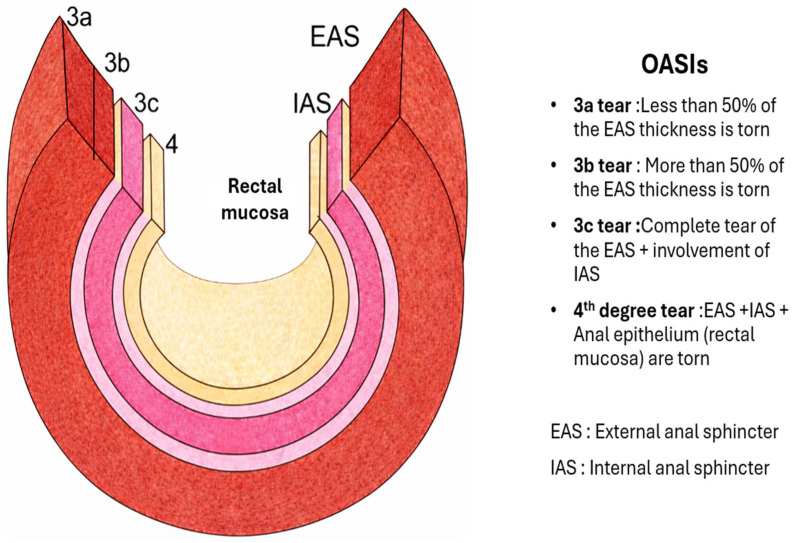
Schematic Representation of Obstetric Anal Sphincter Injury Classification (OASI).

Third- and fourth-degree perineal tears or obstetric anal sphincter injuries (OASIs) are accompanied by high long-term morbidity with anal incontinence, pain, dyspareunia, and adverse outcomes on the quality of life [1,2,3]. OASIs involve disruption of the external anal sphincter (EAS), the internal anal sphincter (IAS), or both, with or without extension to the anorectal mucosa, and represent the leading obstetric cause of anal incontinence in women of reproductive age [1,2]. A schematic representation of OASIs is presented in Figure 1.

The reported incidence of OASIs varies considerably across countries and obstetric settings, ranging from approximately 1% to over 10% of vaginal deliveries, with higher rates consistently observed among primiparous women and instrumental deliveries [2]. Nordic countries and the United Kingdom have reported rates exceeding 5–6%, whereas lower rates are often described in regions where systematic detection may be less rigorous. Importantly, the increasing incidence reported over recent decades is believed to reflect improved recognition, standardized classification systems, and greater clinical awareness rather than a true rise in sphincter trauma alone [2,4]. This epidemiological variability highlights both differences in obstetric practice and the persistent problem of underdiagnosis.

Although careful visual inspection and digital rectal examination (DRE) are mandatory after vaginal birth, clinically occult injuries remain frequently underdiagnosed, contributing to preventable disability [3,5]. Sonographic studies have demonstrated that a substantial proportion of primiparous women with apparently normal clinical examination findings harbor previously unrecognized sphincter defects [6,7]. Guzmán Rojas et al. reported a notable prevalence of anal sphincter defects identified by ultrasound following first vaginal delivery, even in the absence of a clinically diagnosed OASI [6]. Such occult injuries are clinically relevant because delayed or inadequate repair has been associated with the subsequent development of anal incontinence and pelvic floor dysfunction [3,8]. Importantly, symptoms may not manifest immediately postpartum but may develop years later, especially after subsequent vaginal deliveries or age-related pelvic floor changes [8].

Beyond physical morbidity, OASIs impose significant psychological and social burdens. Anal incontinence has been associated with embarrassment, social withdrawal, anxiety, sexual dysfunction, and diminished quality of life [3,5]. The chronic nature of these symptoms may necessitate ongoing medical evaluation, pelvic floor rehabilitation, and occasionally secondary surgical interventions [3]. Consequently, OASIs represent not only a clinical issue but also a broader women’s health concern with substantial long-term implications.

Professional societies such as the Royal College of Obstetricians and Gynaecologists (RCOG) and the American College of Obstetricians and Gynecologists (ACOG) emphasize systematic post-delivery inspection, digital rectal examination, accurate classification of perineal tears, and meticulous repair, followed by structured follow-up when indicated [1,2]. Nevertheless, despite these recommendations, clinically occult sphincter injuries are still reported, suggesting that clinical examination alone may not be sufficient in all cases [5,9].

This gap between recognized underdiagnosis and the limitations of clinical examination has stimulated growing interest in adjunctive imaging modalities. Transperineal ultrasound (TPUS) and endoanal ultrasound (EAUS) allow direct visualization of the anal sphincter complex and provide objective structural assessment beyond digital palpation [9,10,11,12,13,14,15,16]. EAUS is widely regarded as the anatomical reference standard for detailed delineation of sphincter defects [7,10,11,12] and demonstrates particular diagnostic precision for internal anal sphincter involvement [6,13,14]. In contrast, TPUS offers a non-invasive, bedside-accessible alternative with increasing evidence supporting its diagnostic performance, particularly for external anal sphincter injuries [4,13,14,15,16].

In recent years, pelvic floor ultrasound has become progressively integrated into gynecologic and urogynecologic practice, supported by standardized terminology initiatives developed by the International Continence Society (ICS) and the International Urogynecological Association (IUGA) [17]. Furthermore, recent comparative studies and meta-analyses evaluating clinical examination, TPUS, and EAUS have provided deeper insight into the strengths and limitations of each modality in the postpartum setting [18,19]. These developments highlight the need for a contemporary synthesis of available evidence to clarify the role of ultrasound in the diagnosis, management, and follow-up of OASIs.

## 2. Literature Search and Study Selection

This is a structured narrative review with a predefined search strategy, with a descriptive interpretation and synthesis of evidence, without a formal risk-of-bias assessment. We focused on the postpartum diagnosis and management of obstetric anal sphincter injuries (OASIs) from a gynecologic perspective. A comprehensive literature search was performed in PubMed/MEDLINE, Scopus, EMBASE, and the Cochrane Library for studies published between January 2014 and January 2025.

The search strategy included the following keywords and their combinations: “obstetric anal sphincter injury”, “OASI”, “endoanal ultrasound”, “transperineal ultrasound”, “3D ultrasound”, “postpartum”, “classification”, “repair”, “continence”, and “training”. Reference lists of relevant articles and recent reviews were also screened to identify additional eligible studies, including recent advances (2023–2025).

Inclusion criteria were: (i) randomized controlled trials, cohort studies, systematic reviews, and meta-analyses; (ii) studies evaluating transperineal ultrasound (TPUS) and/or endoanal ultrasound (EAUS) in the context of OASIs; and (iii) articles published in English. Exclusion criteria included: (i) case reports, editorials, and conference abstracts; (ii) studies not focusing on obstetric populations; and (iii) studies lacking clear diagnostic or clinical outcome data.

**Figure 2:**
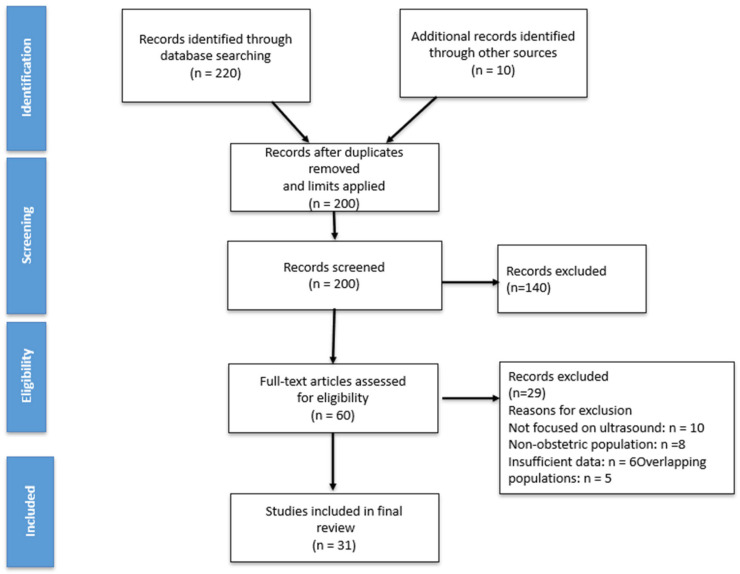
Flow diagram of literature search and study selection for inclusion in the narrative review.

Study selection was performed through screening of titles and abstracts followed by full-text assessment. In cases of overlapping study populations, the most comprehensive and methodologically rigorous report was included. A flow diagram summarizing the study selection process is presented in Figure 2.

## 3. Pathophysiology, Classification, and Burden

OASIs include both third-degree tears (3a: <50% EAS, 3b: >50% EAS, 3c: EAS and IAS), and fourth-degree tears (EAS/IAS with anorectal mucosa), with RCOG- and ACOG-approved systems for classification [1,2]. Long-term sequelae—particularly anal incontinence—may present years after the initial event and they are related to the extent of the defect, levator disruption, and future obstetric event [3,18]. Overlooked IAS disruption is increasingly identified as causingchronic symptoms, further supporting the use of imaging beyond classical digital rectal examination [8,12,19]. The RCOG Green-top Guideline No. 29 [2] and the ACOG Practice Bulletin No. 198 [1] are landmark guidelines for prevention, detection, and repair, recommending early identification, expert repair, and follow-up by specialist perineal units.

The pathophysiology of OASIs reflects the complex biomechanical forces exerted on the pelvic floor during vaginal delivery. Excessive stretching of the perineal tissues during the second stage of labor may result in disruption of the anal sphincter complex, especially when unfavorable conditions such as instrumental delivery, fetal macrosomia, occiput posterior position, or prolonged second stage are present [4,13]. Damage may involve not only the muscular components of the anal sphincter but also associated neural structures, contributing to long-term functional impairment [3]. In particular, disruption of the IAS is increasingly recognized as a critical determinant of passive fecal continence, and injuries to this structure may remain clinically silent at the time of delivery but may manifest later as symptoms of anal incontinence [12,19]. Studies utilizing ultrasound imaging have demonstrated that a considerable proportion of women with apparently intact perineum on clinical examination may harbor occult sphincter defects, highlighting the limitations of visual inspection and digital rectal examination alone [6,9]. Furthermore, the burden of OASIs extends beyond physical morbidity, as persistent symptoms may substantially affect psychological wellbeing, sexual function, and overall quality of life [3,5]. These findings emphasize the importance of accurate early diagnosis and structured follow-up in order to mitigate the long-term consequences associated with these injuries.

## 4. Imaging Modalities: Principles and Practical Issues

### 4.1. Endoanal Ultrasound (EAUS)

Endoanal ultrasound (EAUS) offers high-resolution axial and coronal imaging of the IAS and EAS with the ability for accurate delineation of location, arc length, and depth of defects and scar tissue [8,10]. Three-dimensional (3D) EAUS adds volumetric capability and reproducibility with benefits for classification and surgery planning [10]. Despite these benefits, routine labour ward EAUS is not easy: specialized probes, trained staff, and patient cooperation in the early post-delivery period are needed. Furthermore, the quality of images obtained may be compromised by postpartum pain and oedema [7,20]. In 2023, an initial feasibility study showed that 3D-EAUS used in “screening-like” conditions may add limited diagnostic power over clinical exam alone, most likely due to barriers of implementation rather than an intrinsic deficiency in diagnostic capability [20].

**Figure 3:**
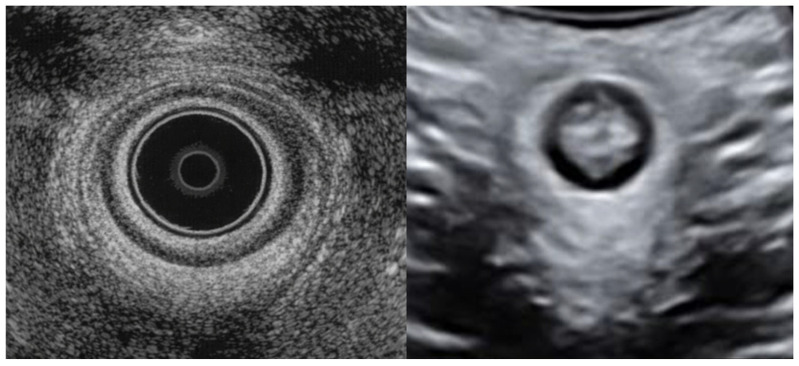
Representative endoanal (EAUS) and transperineal (TPUS) ultrasound images demonstrating normal anal sphincter anatomy.

**Figure 4:**
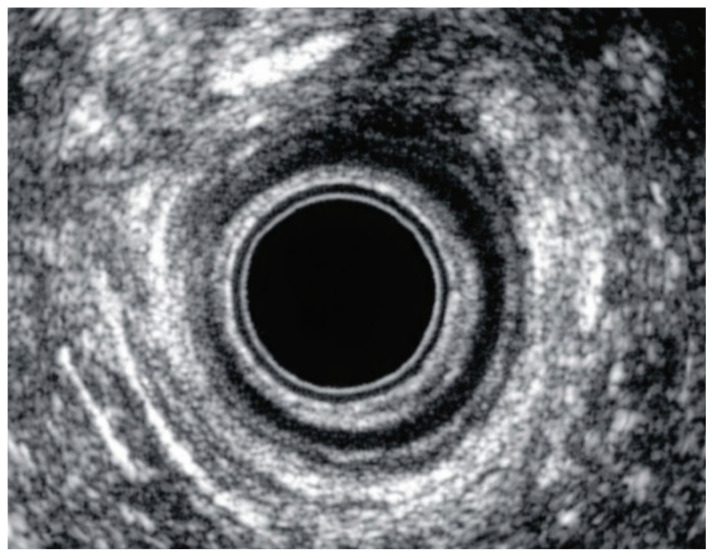
EAUS imaging of an external anal sphincter defect.

Nevertheless, EAUS retains major value in selected cases because of its superior anatomical detail, especially when internal anal sphincter injury is suspected or when precise postoperative mapping of residual defects is required [7,10,11]. In specialist settings, its ability to define complex or multi-level sphincter trauma may improve classification consistency and support decisions regarding re-repair, follow-up intensity, and counseling for future pregnancy [8,10,12]. Thus, although less practical as a universal immediate postpartum screening tool, EAUS remains central as a second-line or reference imaging method in women with equivocal findings, persistent symptoms, or suspected severe injury [7,20]. A representative EAUS ultrasound image demonstrating normal anal sphincter anatomy is presented in Figure 3; an external anal sphincter defect on EAUS is shown in Figure 4.

### 4.2. Transperineal Ultrasound (TPUS)

Transperineal ultrasound (TPUS) is non-invasive, bedside-friendly, and well tolerated. It depicts the anal canal and perineum externally and can be performed by obstetric/gynaecologic sonographers familiar with pelvic floor scanning [4,9,11,14,21]. Recent evidence demonstrates excellent concordance with EAUS for EAS defects and relevance in the immediate postpartum period and follow-up [4,8,9,11,12,13]. Systematic reviews of intrapartum 2D-TPUS reaffirm its intrapartum decision-making function and early identification of the OASI. An additional practical strength of TPUS is that it can be incorporated into routine pelvic floor ultrasound assessment without major patient discomfort or the need for dedicated endoanal equipment [9,14]. This facilitates repeated examination during follow-up and may be particularly useful in busy maternity units or perineal clinics where rapid triage is needed [4,11]. Although TPUS is generally less accurate than EAUS for subtle IAS defects, its accessibility, acceptability, and good performance for EAS trauma make it a pragmatic first-line imaging technique, particularly when clinical findings are uncertain or when immediate postpartum reassessment is required [4,11,13,15,21].

In clinical practice, TPUS may be considered a first-line imaging modality, particularly for assessment of external anal sphincter (EAS) defects and in the immediate postpartum setting [4,9,11,13,14]. However, endoanal ultrasound (EAUS) is preferred in cases of suspected internal anal sphincter (IAS) involvement, equivocal findings, or when detailed anatomical characterization is required for surgical planning and follow-up [2,8,10,12]. A representative TPUS ultrasound image demonstrating normal anal sphincter anatomy is presented in Figure 3.

## 5. Diagnostic Accuracy and Comparative Performance

Across diverse studies, ultrasound detects an appreciable proportion of occult OASIs in primiparas with normal clinical examination [4,5,22]. TPUS has been reported to have sensitivity of up to ~78% for external anal sphincter (EAS) defects, while EAUS may show lower sensitivity for EAS in the acute setting but remains superior for detecting internal anal sphincter (IAS) involvement and for detailed anatomical classification [4,10,11,12,14]. A recent meta-analysis (2025) comparing diagnostic approaches suggests improved diagnostic performance of imaging compared with clinical examination for OASI detection, highlighting the trade-offs between practicality and anatomical detail for TPUS and EAUS [16]. The reliability of clinical examination alone appears limited when compared with standardized imaging approaches, supporting the role of ultrasound in this setting [17].

Advanced 3D/4D imaging techniques may improve spatial visualization and interobserver agreement, particularly for complex or multi-sphincter lesions [10,12]. Notably, the most recent feasibility study questioned the extra diagnostic advantage of universal 3D-EAUS screening early after birth in routine real-world maternityward conditions, owing to education and quality-of-image issues; this underscores the importance of context, operator expertise, and workflow but not the efficacy of EAUS itself [20]. An overview of studies comparing the diagnostic performance of TPUS with that of EAUS is presented in Table 1.

## 6. Clinical Applications in Gynecology

An overview of clinical applications of TPUS and EAUS in OASIs is presented in Table 2.

### 6.1. Immediate Postpartum Detection and Triage

Early postpartum ultrasound, either TPUS or EAUS, can reveal missed defects, expedite the decision to revise repairs, and guide referral to specialist perineal clinics [3,7,12,13,14]. Observational and interventional data suggest ultrasound-supported pathways may facilitate earlier and improved-quality repair and may be associated with reduced rates of anal incontinence, although evidence remains limited and heterogeneous [7,13,14,23]. A Cochrane review previously indicated that EAUS before repair could reduce severe incontinence at 6 months, though more trials are needed [23]. Later on, real-world feasibility evidence emphasizedthe need for structured training to realize these benefits at scale [11].In addition, immediate postpartum imaging may improve diagnostic confidence in cases where edema, bleeding, or pain obscures the clinical examination, particularly after instrumental delivery or when the degree of perineal trauma remains uncertain [7,11,20]. A pragmatic triage approach may therefore involve TPUS as an accessible first-line test in the delivery suite, followed by EAUS when more detailed assessment of sphincter anatomy is required or when internal anal sphincter injury is suspected [12,13,14]. Such an approach may help identify women who would benefit from early specialist review and more intensive postpartum surveillance [3,23].

### 6.2. Post-Repair Evaluation and Follow-Up

TPUS and EAUS define residual defects and scarring of the morphology after primary repair, with correlation to symptoms and anorectal function [12,13,14]. Imaging directs pelvic floor therapy, the need for delayed secondary repair or sphincteroplasty, and counseling concerning future pregnancy [11,12,18]. Follow-up imaging is particularly valuable because anatomical healing after primary repair may not always correspond to symptom severity, and clinically occult residual defects may persist despite apparently satisfactory repair [12,14,18]. In this setting, ultrasound findings can be integrated with continence symptoms and functional testing to support individualized management, including referral for physiotherapy, biofeedback, or colorectal consultation when needed [18]. Moreover, documentation of residual sphincter defects may provide an objective basis for counseling women regarding subsequent pregnancy and mode of delivery, especially when symptoms remain bothersome or imaging demonstrates combined EAS and IAS involvement [8,18].

### 6.3. Predicting and Avoiding Risks

Ultrasound is a valuable tool for prevention by enabling more personalized risk assessment. Perineal body length and anovaginal distance have been associated with an increased risk of OASI [24,25]. Interventional strategies, such as episiometer-guided mediolateral episiotomy during instrumental delivery, have shown potential protective effects compared with routine practice [26,27]. In addition, intrapartum transperineal ultrasound (TPUS) can assist in the assessment of fetal head position and station, potentially reducing the risk of uncontrolled perineal trauma during operative vaginal delivery [28]. These observations support the concept that ultrasound may contribute not only to diagnosis but also to prevention. Antenatal identification of women with shorter perineal body length or reduced anovaginal distance may help define a subgroup at increased risk of severe perineal trauma, allowing more individualized intrapartum management [24,25]. Similarly, intrapartum TPUS may improve the assessment of fetal head descent and malposition, thereby informing decisions regarding operative delivery and episiotomy angle [26,27,28].

Although several predictive models for OASI have been proposed based on clinical and obstetric risk factors, these models remain heterogeneous and lack widespread external validation. Importantly, no established predictive model incorporating ultrasound findings is currently available, representing a key limitation of the current evidence. Overall, while preventive strategies still require validation in larger studies, the available evidence suggests that imaging-informed obstetric care may reduce avoidable sphincter trauma and improve selection of women most likely to benefit from protective interventions [27,28,29].

**Table 2:** Clinical applications of TPUS and EAUS in OASIs: indications, timing, benefits, and limitations.

Clinical Scenario	Preferred Modality	Primary Purpose	Timing	Benefits	Limitations	Key Refs
Immediate post-partum triage (high-risk or uncertain clinical exam)	TPUS first-line; EAUS if unclear	Detect occult OASI; refine grade; guide management	Within 24–72 h after delivery	Bedside; well tolerated; improves detection	Operator dependent; postpartum pain/edema may limit EAUS	[3,8,9,15,23,25]
Pre-repair or specialist assessment	EAUS (3D) ± TPUS	Define extent and depth of defect and sphincter involvement	Early postpartum within 2–12 weeks	High anatomic precision and reproducible	Specialized equipment and expertise needed	[3,8,22]
Post-repair evaluation and follow-up	TPUS or EAUS (3D if complex)	Detect residual defects/scar; guide therapy and prognosis	6–12 weeks postpartum or as needed	Correlates anatomy with function; supports management decisions	Inter-observer variability; Limited standardization	[3,5,8]
Counseling for future delivery	EAUS (IAS/EAS integrity) ± TPUS	Risk stratification; support shared decision-making	Pre-conception or antenatal	Personalized counseling	No standardized criteria; clinical correlation needed	[5,21,27]
Intrapartum decision support	TPUS (2D)	Assess fetal head position/station; anticipate OASI risk	Intrapartum	May reduce uncontrolled perineal trauma	Heterogeneous evidence; operator training needed	[2,16]
Risk prediction (antenatal assessment)	TPUS/perineal measurements	Measure perineal body length; ano-vaginal distance	Antenatal	Identifies high-risk patients	No validated predictive model; not for routine screening	[4,6]

EAS: external anal sphincter; EAUS: endoanal ultrasound; IAS: internal anal sphincter; OASI: Obstetric anal sphincter injuries; TPUS: transperineal ultrasound.

### 6.4. Counseling on the Mode of Future Delivery

The imaging findings (degree of defect, involvement of the IAS), symptoms and manometry can collectively facilitate joint decisions on mode of delivery after OASI [18]. This aligns with RCOG/ACOG guidance to offer customized counseling and documentation [1,2].

In clinical practice, the integration of structural imaging findings with functional assessment allows clinicians to stratify the risk of recurrent sphincter injury and potential deterioration of continence following a subsequent vaginal delivery [8,18]. Women with persistent defects on imaging, particularly when involving the internal anal sphincter or when associated with significant symptoms of anal incontinence, may benefit from individualized discussion regarding the potential advantages of elective cesarean delivery [8]. Conversely, women with intact sphincter anatomy on ultrasound and minimal or absent symptoms may reasonably consider vaginal birth in future pregnancies following appropriate counseling [8,18].

Importantly, in counseling additional obstetric factors should be considered, including fetal size, anticipated mode of delivery, and the presence of other pelvic floor disorders [27,29]. Shared decision-making between the patient and the multidisciplinary clinical team is therefore essential, ensuring that women are adequately informed about both the potential risks of recurrent obstetric anal sphincter injury and the implications of cesarean delivery for future reproductive health [1,2]. In this context, ultrasound evaluation provides an objective anatomical framework that can support balanced and individualized counseling strategies in subsequent pregnancies [9,14].

## 7. Strengths and Limitations of TPUS and EAUS

The primary advantage of TPUS and EAUS is the capability to improve the accuracy of diagnosis further than with clinical examination alone. Both methods are non-invasive, safe, and have the capability of offering dynamic imaging on a real-time basis. TPUS has the additional advantages of easy accessibility, patient acceptability, and practicability of use in the labour ward. Beyond trauma detection, TPUS is widely applied in assessing pelvic floor disorders, including prolapse and urinary incontinence, which frequently coexist with sphincter injuries [21,29,30]. EAUS, particularly the three-dimensional version, has no substitute for precise detailed anatomical classification of the advanced sphincter injury, helping surgical planning as well as providing prognostication.

However, several limitations prevent their wide use. Both modalities rely on the operator, who will require systematic training and expertise to achieve accurate results. Learning curves associated with pelvic floor ultrasound interpretation should also be considered. Studies suggest that diagnostic reliability improves substantially with structured training and standardized acquisition protocols, highlighting the importance of dedicated educational programs for clinicians involved in postpartum perineal assessment [15]. Interobserver variability still persists, despite advances in technology, and standardized acquisition and reporting protocols remain under development [8,10,15,20]. Availability of the EAUS unit, along with patient inconvenience and administrative challenges, makes it impracticable for routine use postpartum in many maternity units. Financial issues and scarcity of thorough health economic assessments also prevent wider use. While professional bodies like the RCOG and the ACOG emphasize the role of careful diagnosis and follow-up, they avoid the recommendation of routine ultrasound screening postpartum, citing the lack of evidence [1,2].

## 8. Implementation: Quality, Service, and Training Models

Training curricula, competency mapping, and Quality Assurance protocols are essential for scaling ultrasound for OASI demands. The following training models have been proposed: (1) Selective pathways model: ultrasound in predefined high-risk scenarios (instrumental delivery, shoulder dystocia, midline episiotomy, severe lacerations on clinical examination, prolonged second stage, occiput posterior position). (2) Perineal clinic model: planned postnatal imaging between 2–12 weeks to detect residual defects that may affect continence outcomes and long-term planning of birth. (3) Labor-ward consult model: on-call sonography/EAUS if suspicion is strong or clinical findings are unclear (staffing and planning of equipment).

**Figure 5:**
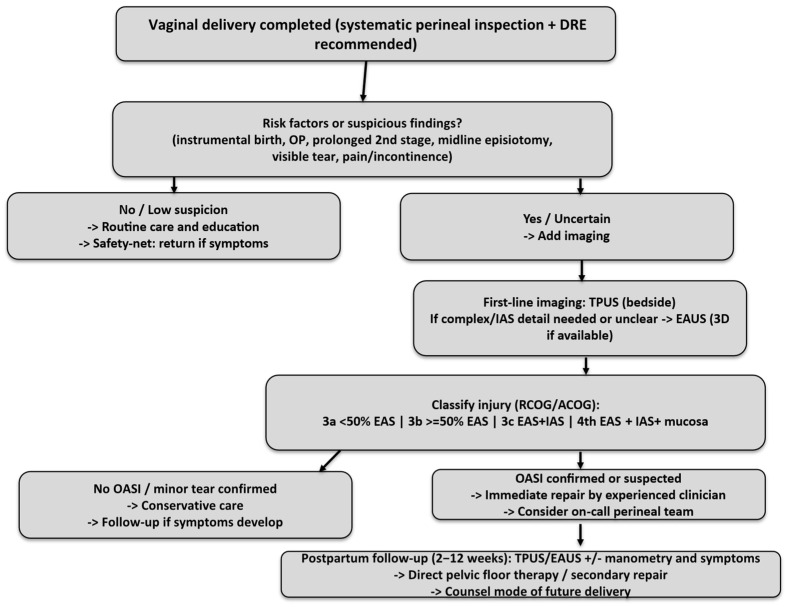
Proposed clinical algorithm for the diagnosis and management of obstetric anal sphincter injuries (OASIs), based on synthesis of current evidence and international guidelines (RCOG, ACOG); not externally validated.

As feasibility studies demonstrate, unless dedicated training and workflowsarein place, routine post-natal EAUS may not do any better than a clinical exam; thus investment in people and process is just as valuable as equipment [20]. A proposed clinical algorithm based on synthesis of current evidence and international guidelines (not externally validated) is represented in Figure 5. This algorithm reflects a pragmatic synthesis of heterogeneous evidence and expert interpretation rather than a validated clinical pathway.

## 9. Future Directions

In the future, several developments will impact the role of ultrasound in the evaluation of OASIs. Standardization of definitions and improvement of interrater reliability between TPUS and 3D-EAUS persist, with current studies aiming to develop robust postpartum and delayed follow-up applicable diagnostic methods [15,31]. Volumetric 3D and 4D TPUS will become more available, with multimodal correlation by combining 3D-EAUS with MRI, potentially clarifying which combinations of imaging modalities result in the very best classification and prognosis [10,12,32]. Beyond ultrasound, decision-support tools are emerging, although current evidence remains preliminary: a recent multicenter trial demonstrated that machinelearning-supported impedance spectroscopy reached diagnostic efficacy similar to that of 3D-EAUS, promising that someday artificial-intelligence-based tools may have the potential to augment sonographic and clinical assessment in the future; however, validation in clinical settings is still required [33]. In parallel, increasing attention is being directed toward the integration of ultrasound imaging into standardized postpartum care pathways. Advances in portable ultrasound systems and automated image analysis may facilitate broader adoption in routine obstetric practice, particularly in high-volume maternity units. Furthermore, combining imaging data with clinical variables and obstetric risk factors may allow the development of predictive models capable of identifying women at increased risk of long-term pelvic floor dysfunction. Such approaches may ultimately support more personalized postpartum surveillance strategies and targeted preventive interventions [31,32,33]. In the end, the field necessitates large, pragmatic randomized controlled trials of selective versus universal postpartum ultrasound techniques, with cost-effectiveness analyses stratified by risk factor and health system. Such evidence will be important to inform potential future changes in international guidelines and will be decisive if ultrasound will become routine or remain concentrated on high-risk individuals.

## 10. Discussion

Obstetric anal sphincter injuries (OASIs) remain a significant source of maternal morbidity despite advances in obstetric care. Although clinical examination after delivery is the standard method for detecting perineal trauma, a substantial proportion of sphincter injuries remain unrecognized at the time of delivery [3,4,5,6]. Missed injuries may lead to persistent structural defects and subsequent anal incontinence, pelvic floor dysfunction, and reduced quality of life, highlighting the need for improved diagnostic strategies.The available evidence is heterogeneous and predominantly based on observational studies, which limits the strength of causal inferences.

Ultrasound-based imaging modalities have emerged as valuable tools for evaluating the anal sphincter complex. Endoanal ultrasound (EAUS) remains the anatomical reference standard, particularly for detailed assessment of internal anal sphincter involvement [8,10,31,32]. However, its use in routine postpartum practice is limited by the need for specialized equipment, trained operators, and patient discomfort in the early postpartum period [7,20].

In contrast, transperineal ultrasound (TPUS) represents a non-invasive and accessible alternative that can be performed at the bedside. Evidence suggests good diagnostic agreement between TPUS and EAUS for external anal sphincter injuries, supporting its role as a practical first-line imaging modality in the immediate postpartum setting [4,9,11]. A stepwise approach—using TPUS initially, followed by EAUS in cases of uncertainty or suspected complex injury—appears to be a pragmatic clinical strategy.

The clinical interpretation of imaging findings should consider the relationship between structural abnormalities and functional outcomes. Not all sphincter defects detected by ultrasound result in symptomatic anal incontinence, as symptom severity is influenced by multiple factors, including neuromuscular integrity and pelvic floor support [18]. Therefore, imaging findings should be integrated with clinical symptoms and functional assessment to guide management decisions.

The timing of imaging is also important, as structural abnormalities identified immediately postpartum may evolve during the healing process. Early imaging should be complemented by structured follow-up, particularly in specialized perineal clinics, to allow correlation between anatomical findings and clinical outcomes over time.

Implementation of ultrasound in obstetric practice requires careful consideration. Current evidence does not support universal postpartum screening, and professional societies recommend selective imaging in high-risk or clinically uncertain cases [1,2]. Furthermore, the effectiveness of ultrasound-based strategies depends on adequate training, standardized protocols, and appropriate clinical pathways.

## 11. Conclusions

Ultrasound plays an increasingly important role in the diagnosis and management of OASIs. When applied selectively and integrated with clinical assessment, TPUS and EAUS can improve detection, guide management, and support individualized counseling regarding future pelvic floor outcomes. From the gynecologic perspective, TPUSand EAUS appear to be useful, adjunctive tools for the prevention, diagnosis, and treatment of OASIs. By disclosing occult damages, assisting with repairs, and providing input on follow-up, these techniques enhance clinical care and decrease late morbidity.

Present evidence supports selective utilization during and after high-risk deliveries and situations of diagnostic doubt, where the risk–benefit ratio and feasibility are highest. However, important knowledge gaps remain. Although ultrasound improves detection of sphincter injuries, high-quality prospective studies evaluating its impact on long-term outcomes and cost-effectiveness are limited. In addition, while predictive models based on clinical factors have been proposed, no validated model incorporating ultrasound findings is currently available. As technology, expertise, and standardization improve, the utilization of ultrasound will probably increase, the desired endpoint being the improvement of maternal care by timely diagnosis and effective treatment of these still underdiagnosed obstetric traumas.

## Figures and Tables

**Table 1:** Diagnostic performance of TPUS vs. EAUS across selected studies.

Study	TPUS	EAUS	Notes
Wong et al. 2022 [13]	TPUS: 78%	EAUS: 59%	TPUS showed higher sensitivity for detecting EAS tears after OASI.
Volløyhaug et al. 2020 [5]	TPUS: 70%(EAS), 43% (IAS)	Not compared directly	TPUS less sensitive for IAS tears but with high specificity.
Dietz et al. 2018 [10]	TPUS: 71–98%	Considered gold standard	TPUS promising for EAS; lower for IAS; EAUS remains diagnostic standard.
Ros et al. 2017 [16]	—	—	Postpartum 2D/3D ultrasound evaluation; agreement with EAUS (add metrics).

EAS: external anal sphincter; EAUS: endoanal ultrasound; IAS: internal anal sphincter; OASI: Obstetric anal sphincter injuries; TPUS: transperineal ultrasound.

## Data Availability

The original data presented in the study are openly available on Medline/PubMed (https://pubmed.ncbi.nlm.nih.gov/, accessed on 25 January 2026), Scopus (https://www.elsevier.com/products/scopus, accessed on 25 January 2026) and the Cochrane Library (https://www.cochranelibrary.com/, accessed on 25 January 2026).

## Abbreviations

The following abbreviations are used in this manuscript:

ACOG	American College of Obstetricians and Gynecologists
EAS	external anal sphincter
EAUS	endoanal ultrasound
IAS	internal anal sphincter
ICS	International Continence Society
IUGA	International Urogynecological Association
OASI	Obstetric anal sphincter injury
RCOG	Royal College of Obstetricians and Gynaecologists
TPUS	transperineal ultrasound

## References

[1] Committee on Practice Bulletins-Obstetrics (2018). ACOG Practice Bulletin No. 198: Prevention and Management of Obstetric Lacerations at Vaginal Delivery. Obstet. Gynecol..

[2] Royal College of Obstetricians and Gynaecologists (RCOG) (2015). Third- and Fourth-Degree Perineal Tears, Management (Green-Top Guideline No. 29).

[3] Taithongchai A., Veiga S.I., Sultan A.H., Thakar R. (2020). Consequences of undiagnosed OASIs. Int. Urogynecol. J..

[4] Seidenari A., Cuicchi D., Youssef A., Oliver E.A., Montaguti E., Bellussi F. (2021). Obstetric anal sphincter injuries: Strategies for prevention, diagnosis, and management. Minerva Obstet. Gynecol..

[5] Volløyhaug I., Taithongchai A., Arendsen L., van Gruting I., Sultan A.H., Thakar R. (2020). Is endoanal, introital or transperineal ultrasound diagnosis of sphincter defects more strongly associated with anal incontinence?. Int. Urogynecol. J..

[6] Guzmán Rojas R.A., Shek K.L., Langer S.M., Dietz H.P. (2013). Prevalence of anal sphincter injury in primiparous women. Ultrasound Obstet. Gynecol..

[7] O’Leary B.D., Kelly L., Fitzpatrick M., Keane D.P. (2023). Underdiagnosis of internal anal sphincter trauma after vaginal delivery. Ultrasound Obstet. Gynecol..

[8] Okeahialam N.A., Thakar R., Sultan A.H. (2021). Subsequent pregnancy after OASI: Sphincter integrity and function. Int. Urogynecol. J..

[9] Bellussi F., Dietz H.P. (2021). Postpartum ultrasound for the diagnosis of OASI. Am. J. Obstet. Gynecol..

[10] Dietz H.P. (2018). Exoanal imaging of the anal sphincters. J. Ultrasound Med..

[11] Tejedor P., Plaza J., Bodega-Quiroga I., Ortega-López M., García-Olmo D., Pastor C. (2019). The Role of Three-Dimensional Endoanal Ultrasound on Diagnosis and Classification of Sphincter Defects After Childbirth. J. Surg. Res..

[12] Kirss J., Huhtinen H., Niskanen E., Ruohonen J., Kallio-Packalen M., Victorzon S., Victorzon M., Pinta T. (2019). Comparison of 3D endoanal ultrasound and external phased array magnetic resonance imaging in the diagnosis of obstetric anal sphincter injuries. Eur. Radiol..

[13] Wong K.W., Thakar R., Sultan A.H., Andrews V. (2022). Can transperineal ultrasound improve the diagnosis of OASIs?. Int. Urogynecol. J..

[14] Dietz H.P. (2023). Diagnosis of maternal birth trauma by pelvic floor ultrasound. Eur. J. Obstet. Gynecol. Reprod. Biol..

[15] Eisenberg V.H., Valsky D.V., Yagel S. (2019). TPUS assessment after OASI. Ultrasound Obstet. Gynecol..

[16] Ros C., Martínez-Franco E., Wozniak M.M., Cassado J., Santoro G.A., Elías N., López M., Palacio M., Wieczorek A.P., Espuña-Pons M. (2017). Postpartum two- and three-dimensional ultrasound evaluation of anal sphincter complex in women with obstetric anal sphincter injury. Ultrasound Obstet. Gynecol..

[17] Doumouchtsis S.K., de Tayrac R., Lee J., Daly O., Melendez-Munoz J., Lindo F.M., Cross A., White A., Cichowski S., Falconi G. (2023). An International Continence Society (ICS)/International Urogynecological Association (IUGA) joint report on the terminology for the assessment and management of obstetric pelvic floor disorders. Int. Urogynecol. J..

[18] Pang J., Gao Y., Yin N., Liu X. (2025). Diagnostic performance of different examination methods for detecting obstetric anal sphincter injuries: A meta-analysis comparing multiple diagnostic methods. Eur. J. Obstet. Gynecol. Reprod. Biol..

[19] Santoro G.A., Pelizzo P., Di Tanna G.L., Grossi U., Castronovo F., Alharbi M., Busato E., Zanus G. (2025). Reliability of clinical examination for the assessment of obstetric anal sphincter injuries. A comparative study with 3D endoanal ultrasonography. Eur. J. Obstet. Gynecol. Reprod. Biol..

[20] Wong K.W., Thakar R., Andrews V., Sultan A.H. (2022). Role for TPUS immediately after primary repair of 3rd-degree tears?. Eur. J. Obstet. Gynecol. Reprod. Biol..

[21] Huber M., Larsson C., Harrysson M., Strigård K., Lehmann J.P., Nordin P., Tunón K. (2023). Use of endoanal ultrasound in detecting obstetric anal sphincter injury immediately after birth. Acta Obstet. Gynecol. Scand..

[22] Walsh K.A., Grivell R.M. (2015). Use of endoanal ultrasound for reducing the risk of complications related to anal sphincter injury after vaginal birth. Cochrane Database Syst. Rev..

[23] Villani F., Cosmi E., Lunardon Z., Granci M., Panizza C., Mazzucato B., Cavalieri A., Toma M.M., Furau R., Furau C. (2024). Antenatal Anovaginal Distance, a Potential Indicator of Perineal Damage during Pregnancy. Healthcare.

[24] Geller E.J., Robinson B.L., Matthews C.A., Celauro K.P., Dunivan G.C., Crane A.K., Ivins A.R., Woodham P.C., Fielding J.R. (2014). Perineal body length as a risk factor for ultrasound-diagnosed anal sphincter tear at first delivery. Int. Urogynecol. J..

[25] Sriram S.N., Dorairajan G., Rane A. (2024). Obstetric Anal Sphincter Injury After Episiometer-Guided Versus Conventional Episiotomy in Instrumental Deliveries: A Randomized Controlled Trial. Int. Urogynecol. J..

[26] DrusanyStaric K., Lukanovic A., Petrocnik P., Zacesta V., Cescon C., Lucovnik M. (2017). Impact of mediolateral episiotomy on incidence of obstetrical anal sphincter injury diagnosed by endoanal ultrasound. Midwifery.

[27] Packet B., Page A.-S., Cattani L., Bosteels J., Deprest J., Richter J. (2023). Predictive factors for obstetric anal sphincter injury in primiparous women: Systematic review and meta-analysis. Ultrasound Obstet. Gynecol..

[28] Nebel S., Vardon D., Dreyfus M., Pizzoferrato A.C. (2025). 2D-transperineal ultrasound in delivery room: Contribution in assessing labor progress, predicting outcome of labor and recognizing obstetric anal sphincter injuries (OASIS). A systematic review. J. Gynecol. Obstet. Hum. Reprod..

[29] Eggebø T.M., Volløyhaug I. (2024). The pelvic floor during pregnancy and delivery: Can trauma/disorders be prevented?. Acta Obstet. Gynecol. Scand..

[30] Mikos Τ., Theodoulidis I., Karalis Τ., Zafrakas M., Grimbizis G.F. (2024). Instruments used for the assessment of SUI severity in urogynecologic surgical trials: A scoping review. Int. Urogynecol. J..

[31] Gräs S., Starck M., Jangö H., Lose G., Klarskov N. (2024). The Reliability of 3-Dimensional Endoanal Ultrasonography Early and Late Postpartum. Urogynecology.

[32] Borycka K., Młyńczak M., Rosoł M., Korzeniewski K., Iwanowski P., Heřman H., Janku P., Uchman-Musielak M., Dosedla E., Diaz E.G. (2025). Detection of obstetric anal sphincter injuries using machine learning-assisted impedance spectroscopy: A prospective, comparative, multicentre clinical study. Sci. Rep..

[33] Sanozidis A., Zafrakas M., Athanasiadis A.P., Papasozomenou P., Assimakopoulos E., Tsolakidis D., Grimbizis G., Mikos T. (2026). Hiatal changes in multiparous women during pregnancy and after delivery: A prospective 3D-transperineal ultrasound study. Clin. Exp. Obstet. Gynecol..

